# Non-Invasive Imaging of Tumors by Monitoring Autotaxin Activity Using an Enzyme-Activated Near-Infrared Fluorogenic Substrate

**DOI:** 10.1371/journal.pone.0079065

**Published:** 2013-11-20

**Authors:** Damian Madan, Colin G. Ferguson, Won Yong Lee, Glenn D. Prestwich, Charles A. Testa

**Affiliations:** 1 Echelon Biosciences Inc., Salt Lake City, Utah, United States of America; 2 Department of Medicinal Chemistry, University of Utah, Salt Lake City, Utah, United States of America; Stanford University, United States of America

## Abstract

Autotaxin (ATX), an autocrine motility factor that is highly upregulated in metastatic cancer, is a lysophospholipase D enzyme that produces the lipid second messenger lysophosphatidic acid (LPA) from lysophosphatidylcholine (LPC). Dysregulation of the lysolipid signaling pathway is central to the pathophysiology of numerous cancers, idiopathic pulmonary fibrosis, rheumatoid arthritis, and other inflammatory diseases. Consequently, the ATX/LPA pathway has emerged as an important source of biomarkers and therapeutic targets. Herein we describe development and validation of a fluorogenic analog of LPC (AR-2) that enables visualization of ATX activity *in vivo*. AR-2 exhibits minimal fluorescence until it is activated by ATX, which substantially increases fluorescence in the near-infrared (NIR) region, the optimal spectral window for *in vivo* imaging. In mice with orthotopic ATX-expressing breast cancer tumors, ATX activated AR-2 fluorescence. Administration of AR-2 to tumor-bearing mice showed high fluorescence in the tumor and low fluorescence in most healthy tissues with tumor fluorescence correlated with ATX levels. Pretreatment of mice with an ATX inhibitor selectively decreased fluorescence in the tumor. Together these data suggest that fluorescence directly correlates with ATX activity and its tissue expression. The data show that AR-2 is a non-invasive and selective tool that enables visualization and quantitation of ATX-expressing tumors and monitoring ATX activity *in vivo*.

## Introduction

**Figure 1:**
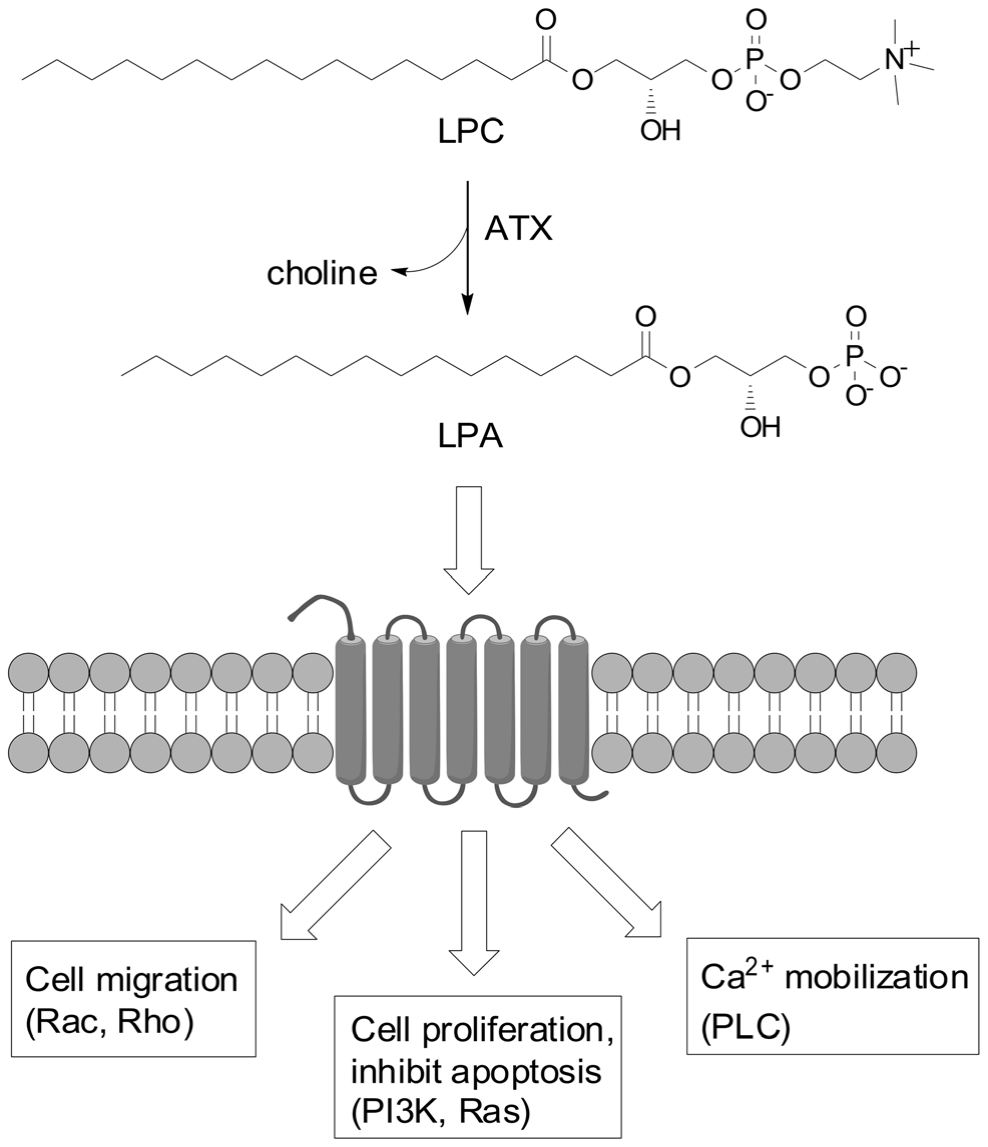
The ATX/LPA pathway. ATX cleaves the lipid LPC to generate LPA. LPA binding to GPCRs stimulates several signaling pathways.

Autotaxin (ATX) and its lipid product lysophosphatidic acid (LPA) are key players in cell proliferation and motility and are thus central to both normal physiology and the physiology of several diseases. ATX is a mammalian extracellular lipase with lyosphospholipase D (lysoPLD) activity that hydrolyzes lysophosphatidylcholine (LPC) to the lysolipid mediator LPA and choline (Figure 1). LPA binds at least six G protein-coupled receptors (GPCRs) to trigger multiple downstream signaling cascades, such as activation of Rho and Ras small GTPases. LPA also activates the nuclear transcription factor peroxisomal proliferating activating receptor (PPARγ). Increased levels of LPA are linked to poor outcomes in ovarian and breast cancer and have been shown to regulate cancer cell proliferation, invasion, angiogenesis, and biochemical resistance to chemotherapy and radiation therapy-induced apoptosis [1]–[3].

Overexpression of ATX contributes to the pathophysiology of invasive cancers [4], [5], and ATX levels are elevated in various cancers such as melanoma, breast cancer, renal cancer, non-small-cell lung cancer, neuroblastoma, hepatocellular carcinoma, glioblastoma multiforme, Hodgkin's lymphoma, and thyroid cancer [6]. Notably, breast cancer cell invasiveness is linked to ATX activity, and forced over-expression of ATX or one of three LPA receptors in the mammary epithelium is sufficient to induce mammary tumorigenesis [7]. The gene encoding ATX (*ENPP2*) is among the 40 most upregulated genes in highly metastatic cancers [8], [9] with lysoPLD activity correlating with poor prognosis [10], [11]. Elevated levels of ATX are also associated with other diseases including multiple fibrotic diseases such as idiopathic pulmonary fibrosis and scleroderma, rheumatoid arthritis, chronic liver disease, obesity, multiple sclerosis, Alzheimer's disease, neuropathic pain, and primary open angle glaucoma [5], [12], [13]. Recently, a strategy to inhibit ATX showed promising therapeutic potential in idiopathic pulmonary fibrosis [14].

Considerable effort is being devoted to develop drugs targeting the ATX/LPA axis with several promising drug candidates such as PF-8380, showing good bioavailability for *in vivo* applications [15], [16]. As ATX inhibitors progress to the clinic, a diagnostic method is needed to measure ATX activity *in vivo*, both during drug development and during ATX-directed therapy. Such a method could (i) visualize ATX activity in preclinical animal models, (ii) identify patients that would benefit from ATX therapy, and (iii) once these patients are enrolled, could serve as a companion diagnostic. Knowing that a candidate ATX inhibitor is active against the target at both a molecular and a tissue level provides critical preclinical and potentially clinical information. Similar personalized medicine approaches are currently used in the clinic for other treatments [17]. Representative examples include HER-2/transtuzumab and BCR-ABL/imatinib. HER-2 expression status is evaluated in breast cancer patients to predict transtuzumab (Herceptin) efficacy, while imatinib (Gleevec) can be prescribed when BCR-ABL testing in chronic myelogenous leukemia patients reveals the presence of the Philadelphia chromosome.

Currently, monitoring the ATX/LPA pathway in animals relies on *ex vivo* measurements of ATX or LPA levels in biological fluids. However, LPA turnover in serum is both rapid and exquisitely sensitive to sample acquisition, thereby limiting the utility of this approach [18]. Moreover, activation of the ATX/LPA axis usually involves localized tissue responses which are not reflected in serum levels [19]. Other methods are indirect, relying on monitoring physiological changes related to the ATX/LPA axis, such as changes to tumor volume [20]. Finally, measuring ATX and LPA levels in harvested tissues limits data collection to an end-point analysis. An experimental tool that permits monitoring ATX activity in three spatial dimensions as well as over a time course during treatment with an ATX inhibitor will significantly advance ATX research and development efforts.

**Figure 2:**
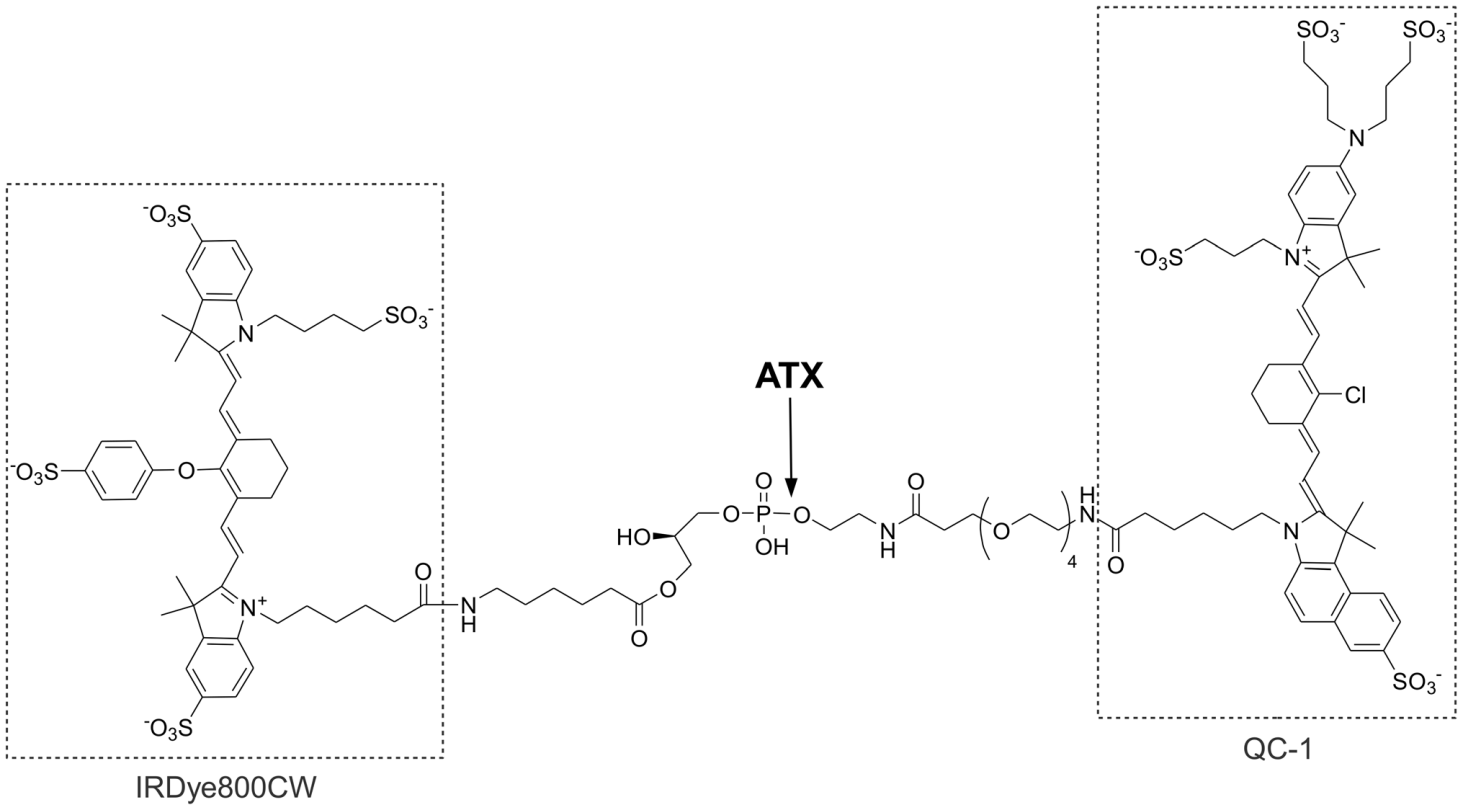
Structure of AR-2.

To address the unmet need to non-invasively measure ATX activity *in vivo* we developed a near-infrared imaging probe, AR-2 (Figure 2). Other imaging probes that target intracellular components require elements that allow the probes to penetrate the cell membrane. Since ATX is a secreted enzyme, these mechanisms were not required for the AR-2 design. AR-2contains a lipid-linked fluorophore and a choline-linked quencher, exploiting the lysoPLD activity of ATX to liberate a near-infrared fluorescence (NIRF) signal upon cleavage by ATX. While visible light poorly penetrates most living systems, NIRF (700–900 nm) is a sensitive imaging modality that has been used to deeply image living tissue [21]. While NIRF imaging has yet to be approved for clinical applications, promising results from several investigational human studies suggest this modality will make the transition into clinic settings shortly [22]

Herein we used AR-2 to visualize ATX activity in a mouse breast cancer model. Fluorescence intensity correlated with ATX levels, and decreased signal was observed when animals were pretreated with an ATX inhibitor. The data demonstrate the utility of AR-2 for *in vivo*, tissue-specific visualization of ATX activity as well as its potential as a companion diagnostic for treatments targeting diseases in which ATX activity is dysregulated.

## Results and Discussion

AR-2 was designed as an analog of LPC, in which a fluor (IRDye® 800CW) was linked to the *sn*-1 acyl group and a quencher (IRDye® QC-1) was appended to a mimic of the choline head group. IRDye® 800CW has optimal excitation and emission maxima for *in vivo* optical imaging (775 nm and 800 nm, respectively) with many imaging platforms tuned to these fluorescence properties, and as a result, has been widely used for basic and translational research programs [23]–[25]. AR-2 is modeled after FS-3, a fluorescent ATX substrate previously validated for monitoring activity and inhibitor screening *in vitro* [6], [26]–[28]. AR-2 was further engineered so that the predicted LPA-like product of ATX-mediated cleavage is fluorescent. The fluorescent product formed was expected to interact with cell surface receptors in a similar fashion to LPA, thereby slowing clearance and increasing signal lifetime [29]–[31].

**Figure 3:**
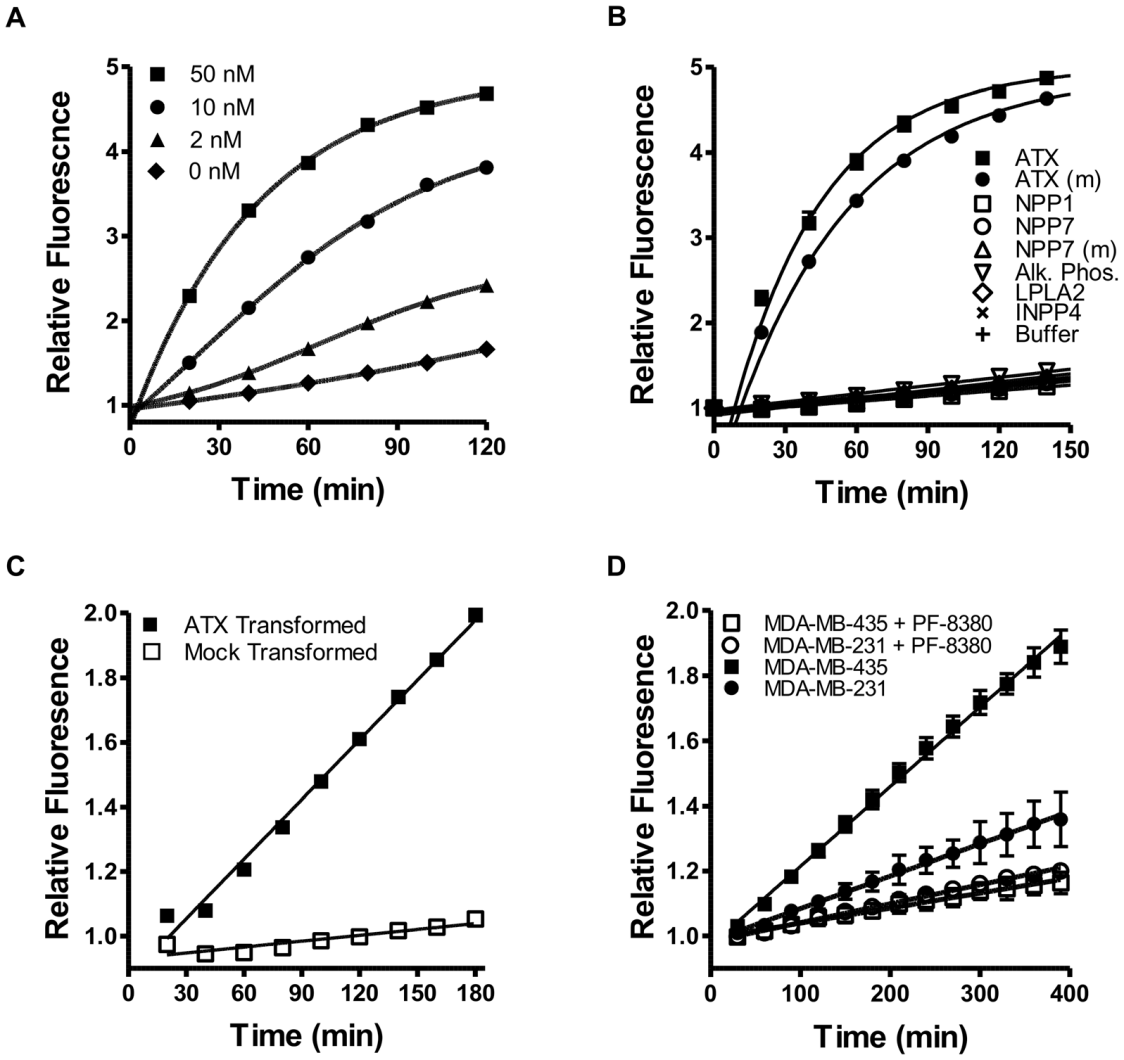
AR-2 is specifically activated by ATX *in vitro*. (**a**) AR-2 was incubated with varying concentrations of purified ATX and fluorescence was measured over time. (**b**) AR-2 was incubated with enzymes homologous to ATX. Enzyme reactions included human and mouse ATX, human NPP1, human and mouse NPP7, shrimp alkaline phosphatase, human LPLA2 and human INPP4A. Mouse enzymes are indicated with (m). (**c**) AR-2 was incubated with media from COS-7 cells transfected with ATX-encoding or mock plasmid and fluorescence was monitored over time. (**d**) AR-2 was incubated with conditioned media from cancer cells overexpressing ATX in the presence and absence of PF-8380 (1 µM), and fluorescence was monitored over time. For all panels results are means ± SD from at least three experiments.

AR-2 was validated as an ATX substrate *in vitro* by monitoring fluorescence with increasing amounts of purified ATX (Figure 3A). Indeed, a strong time-dependent increase in fluorescent signal was observed, and the rate of signal formation was dependent on ATX concentration. Reverse phase HPLC of these samples was consistent with ATX cleaving AR-2 at the predicted site (data not shown) When reactions were performed in the presence of ATX inhibitors PF-8380, S32826, or BrP-LPA, fluorescence signal generation was attenuated with similar inhibition constants to those observed for FS-3 (Table 1). Thus, like FS-3, AR-2 is enzymatically cleaved by the lysoPLD activity of ATX, despite the implications from the ATX crystal structure that the size of the hydrophobic lipid-binding channel would preclude AR-2 from binding and being cleaved [31]–[33]. Indeed, ATX shows very low substrate selectivity. Comparisons between LPC and FS-3 hydrolysis show that kinetic details are likely dependent on the structure of the substrate [34].

**Table 1:** K_i_ of ATX inhibitors calculated using different fluorogenic ATX substrates.

		PF-8380	S32826	BrP-LPA
ATX Substrate	K_m_ (µM)	K_i_ (nM)	K_i_ (nM)	K_i_ (nM)
AR-2	0.66±0.05	3.2±1.0	1.6±1.9	300±300
FS-3	3.7±0.2	7.3±1.5	5.5±3.3	320±70

To be useful *in vivo*, AR-2 must be specifically activated by ATX to avoid competing background signal. To determine selectivity, a panel of homologous enzymes was evaluated for activation of AR-2 (Figure 3B). Human and mouse ATX both activated AR-2 with similar efficiencies, yet none of the related enzymes tested generated a fluorescence response above buffer control, indicating that AR-2 activation is highly specific for ATX *in vitro*.

To evaluate potential ATX-independent activation of AR-2 that might occur *in vivo*, AR-2 was incubated with media from cells overexpressing ATX. First, COS-7 cells were transfected with an ATX-encoding plasmid and compared to mock-transfected cells. As expected, media from ATX-transfected, but not mock-transfected, cells activated AR-2, signifying that neither media components nor factors released from this cell type, other than ATX, efficiently activate AR-2 (Figure 3C).

In a second set of experiments, AR-2 activation was measured in conditioned media from MDA-MB-435 melanoma and MDA-MB-231 breast cancer cell lines, both of which overexpress ATX. In both cases, conditioned media resulted in an increased NIRF signal over time (Figure 3D). Media from MDA-MB-435 cells exhibited the most rapid signal generation, correlating with reported higher ATX expression levels relative to MDA-MB-231 cells [35]–[37]. When PF-8380 was added to either reaction, the rate of signal generation was dramatically reduced. Taken together, these results indicate that ATX specifically activates AR-2 in cell culture systems.

**Figure 4:**
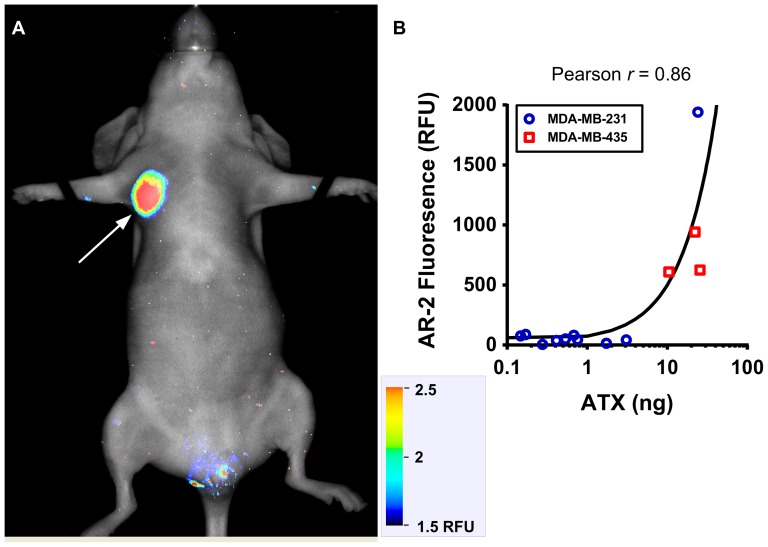
AR-2 fluorescence *in vivo* correlates with ATX levels. (**a**) A mouse bearing an MDA-MB-231 orthotopic tumor (arrow) was injected with AR-2 by tail vein injection and NIRF was imaged. (**b**) AR-2 tumor-bearing mice similar to (a), or bearing an MDA-MB-435 orthotopic tumor were imaged and *in vivo* tumor fluorescence was determined (n = 11 for MDA-MB-231 or n = 3 for MDA-MB-435). Tumors were then excised and ATX levels were measured in tumor homogenates by ELISA. The data were plotted as tumor fluorescence versus ATX mass and fit using linear regression analysis. The slope of the resulting line demonstrates significant correlation (p<0.0001).

To determine whether ATX can activate AR-2 *in vivo*, mice were orthotopically implanted with human MDA-MB-231 breast tumor cells encapsulated in a semi-synthetic extracellular matrix (sECM) hydrogel [35], [37], [38]. Engineered tumors were positioned in the right, upper, thoracic, mammary gland, fat pads. A small cohort (n = 3) of mice were orthotopically implanted with human MDA-MB-435 melanoma cells using the same procedure describe for MDA-MB-231 cells (*in vivo* imaging for the MDA-MB-435 tumors will be reported elsewhere). Once xenotransplanted tumors were established, AR-2 was delivered intravenously, and NIRF was subsequently imaged in whole bodies of live animals. NIRF signals were tumor-specific and elevated compared to the rest of the body (Figure 4A), and in accordance with cell culture data (Figure 3D), MDA-MB-435 tumors showed, on average, higher AR-2 activation than MDA-MB-231 tumors. To confirm that AR-2 activation is dependent on the concentration of ATX, ATX levels were determined in the excised tumors. The ATX concentration in the tissue homogenates strongly correlated with tumor NIRF levels over a large range of intra-tumoral ATX levels (Figure 4B). These data are consistent with a mechanism whereby the fluorescent product produced by ATX-mediated cleavage of AR-2 accumulates where there are higher levels of ATX. We cannot exclude the possibility that AR-2 is activated more globally (*e.g.* in circulation) and the fluorescent product selectively accumulates in the tumor. Nevertheless, there is an increase in NIRF signal for ATX-expressing tumors that correlates with intra-tumoral ATX levels.

The intensity of AR-2 fluorescence in healthy tissues rapidly rises and decays quickly due to the background fluorescence from intact AR-2. The rapid disappearance of AR-2 fluorescence intensity in the body suggests that boosting the signal-to-noise ratio can be achieved by imaging when the majority of AR-2 is eliminated from the body while its metabolite is retained *in situ*. To determine the optimal timing for imaging, we investigated the pharmacokinetic properties of AR-2 in mice. The time course of AR-2 in the plasma after intravenous dosing shows bi-exponential decrease over time (Figure S1). The elimination half-life is around 8.5 h suggesting that approximately 97% of AR-2 is eliminated 42 h after dosing (5 half-lives). This timing is consistent with low background fluorescence in the animals resulting in high signal-to-noise ratio in the tumors when imaged 48 h after dosing.

**Figure 5:**
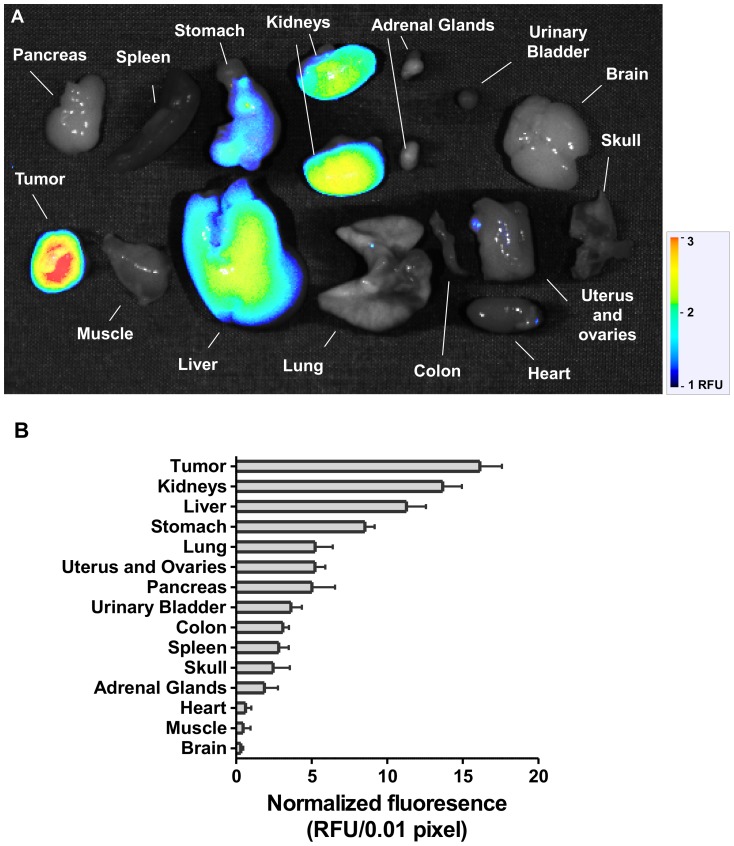
AR-2 tissue distribution. (a) A mouse bearing an MDA-MB-231 xenograft was treated with AR-2. 48 hours later, the mouse was sacrificed and the dissected organs were imaged *ex vivo*. (**b**) Fluorescence intensities normalized for area were compared. Results are means ± SEM from three experiments.

When intravenously administered, AR-2 produces a rapid rise of intense fluorescence signal in the whole body. The apparent volume of distribution (V*_d_*) of AR-2 is 432 mL/kg, *b.w*. (or 8.6 mL in 20 g mouse) close to 43% of body weight suggesting that AR-2 distributes extensively [39]. To investigate how AR-2 distributes and is eliminated from the body, mice bearing MDA-MB-231 xenografts were sacrificed 48 h after AR-2 dosing, then tissues were harvested and the fluorescence signals measured. The NIRF signal in excised tumors was strongly elevated compared to that of most other organs (Figure 5). Moderate signals were present in kidneys and liver suggesting that AR-2 is primarily eliminated by these organs. Modest fluorescence observed in the stomach is likely a result of AR-2 cleavage due to the highly acidic pH. Overall, these data suggested that optimal imaging for ATX in this tumor model is achieved 48 h after intravenous administration of AR-2.

**Figure 6:**
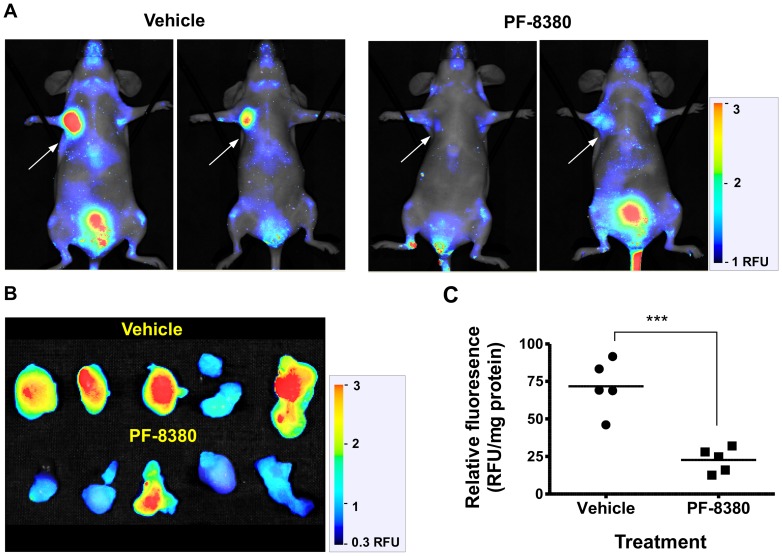
AR-2 is specifically activated by ATX *in vivo* with fluorescence attenuated by addition of an ATX inhibitor. (**a**) Mice bearing MDA-MB-231 xenografts were treated with vehicle or the ATX inhibitor PF-8380. Twenty minutes later, AR-2 was injected *i.v.*, and the mice were subsequently imaged with NIRF. (**b**) Tumors from (a) were imaged *ex vivo*. (**c**) *Ex vivo* tumor fluorescence was normalized to total protein levels (*** denotes p<0.001).

Next, the effect of an ATX inhibitor in tumor bearing mice was investigated to determine whether AR-2 fluorescence would decrease in a targeted fashion in response to the inhibitor. Thus, mice orthotopically implanted with MDA-MB-231 tumors were treated with PF-8380 by a single oral gavage (30 mg/kg) or with vehicle alone 20 min prior to AR-2 injection. High NIRF signals were clearly visible in tumors from vehicle-treated mice, with markedly lower signals observed in the PF-8380-treated group (Figure 6A). To compare the fluorescence intensity in the tumors, fluorescence in the excised tumor was measured and normalized to the quantity of protein (Figure 6). The results were striking, with fluorescence signals in tumors from PF-8380 treated animals significantly reduced (P<0.001) compared to tumors from vehicle-treated mice. Taken together, these data strongly suggest that ATX selectively activates AR-2 *in vivo*, and the fluorescent signal directly correlates with the level of ATX expression.

Here, we describe the first *in vivo* “smart probe” capable of measuring and imaging ATX activity in living organisms. The AR-2 probe is a valuable tool with several potential clinical applications, including (i) discovery and development of pharmacological inhibitors targeting ATX, (ii) identification of ATX-expressing cancers and other ATX-related diseases, (iii) selection of patients who are candidates for ATX inhibitor treatment, (iv) development of a companion diagnostic for ATX-directed therapies, and (v) guiding surgical resection of ATX-expressing tumors. In addition, AR-2 will allow discovery of additional physiological and pathophysiological roles for ATX *in vivo*.

## Methods

### Reagents

Purified human ATX, INPP4A, LPLA2, PF-8380, S32826, BrP-LPA and FS-3 were obtained from Echelon Biosciences, Inc. (Salt Lake City, UT). Mouse ATX, human NPP1, and mouse and human NPP7 were purchased from R&D Systems (Minneapolis, MN). Shrimp alkaline phosphatase was purchased from Promega (Madison, WI). COS-7, MDA-MB-231, and MDA-MB-435 cell lines were purchased from ATCC (Manassas, VA). Extracel was purchased from the Glycosan division of BioTime, Inc (Alameda, CA). IRDye® 800CW and IRDye® QC-1 NHS esters were from LI-COR (Lincoln, NE). Bradford reagent was purchased from Sigma-Aldrich (St. Louis, MO).

### Synthesis of AR-2

AR-2 was synthesized following the steps described for FS-3 substituting the NHS esters of the near infrared dye IRDye® 800CW and quencher IRDye® QC-1 [26]. The product was 95% pure by HPLC. Detailed procedures and characterization data can be found in Figure S2.

### AR-2 reactions with purified enzymes

AR-2 (100 nM) was incubated with the indicated enzyme in buffer containing 50 mM Tris-Cl pH 8.0, 5 mM KCl, 1 mM CaCl_2_, 1 mM MgCl_2_, 140 mM NaCl, and 1 mg/mL fatty acid free bovine serum albumin. Reactions were immediately placed in a Spectramax M2 plate reader (Molecular Devices, Sunnyvale, CA) set at 37°C, monitoring fluorescence exciting at 750 nm and emitting at 805 nm over time. Unless otherwise stated, all reactions used 10 nM enzyme, except alkaline phosphatase, which was performed at 0.1 unit/µL.

### Evaluation of ATX inhibition

To test ATX inhibition, stock solutions of PF-8380, S32826 or BrP-LPA were prepared in DMSO then diluted with water to achieve the final experimental concentrations. DMSO was spiked into each reaction mixture to equalize vehicle concentrations. Purified human ATX (2 nM final concentration) was mixed with varying concentrations of inhibitor at 37°C for 10 min, then AR-2 or FS-3 was added to a final concentration of 1 µM. The rates of fluorescence increase were measured for up to 2 h. The initial velocity of fluorescence increase was determined and normalized to control reactions. The IC_50_ of each compound was calculated by fitting a standard curve using a sigmoidal dose-response (variable slope) nonlinear regression curve using GraphPad Prism (version 5.03).

The Michaelis constant (K_m_) was determined for each compound by measuring the rate of fluorescence increase at varying substrate concentrations using Prism to fit to the Michaelis-Menton equation. Inner filter effects were overcome using previously described methods [40]. The inhibition constant (K_i_) for each set of reactions was determined from the IC_50_ following standard methods.

### Cell culture

COS-7 cells grown in Dulbecco's Modified Eagle Medium (DMEM) supplemented with 10% FBS and penicillin/streptomycin were transfected with human β ATX cDNA inserted into pcDNA DEST40 (Invitrogen, CA) or mock plasmid in DMEM without FBS using TransIT®-2020 transfection reagent (Mirus Bio, WI) according to the manufacturer's protocol.

MDA-MB-231 and MDA-MB-435 cells were grown to 80% confluence in Roswell Park Memorial Institute (RPMI) medium supplemented with 10% FBS. Cells were rinsed twice with PBS and grown for approximately 60 h in RPMI supplemented with 1% FBS. Media were centrifuged at 1000× *g* for 1 min to collect cells. Conditioned media was prepared by concentrating the supernatant ∼40-fold using Amicon Ultra YM30 (Millipore, Billerica, MA).

Equal volumes of cell media (COS-7) or conditioned media (MDA-MB-231 or MDA-MB 435) were mixed with buffer to achieve a final concentration of AR-2 (1 µM). All other conditions were as described for purified enzymes.

### Xenograft mouse tumor model

Animal studies were approved by the Institutional Animal Care and Use Committee (IACUC) of the University of Utah (protocol numbers 10-10016 and 12-05013). Tumors in mice were established as previously described [20], [41]. Briefly, approximately ten-week old female nude (Nu/Nu) mice (Charles River Laboratories International, Wilmington, MA) were lightly anesthetized with isoflurane and subcutaneously inoculated with MDA-MB-231 cells in the left thoracic mammary glands. Prior to inoculation, cells were suspended in Extracel to make 5×10^7^ cells/mL and each mouse received 0.1 mL of Extracel tumor suspension. To reduce auto-fluorescence arising from the feed, mice were switched to specially formulated diet (2920X, Harlan Laboratories, WI) at least two days prior to imaging.

### *In vivo* imaging

Six weeks after tumor graft, mice were imaged using AR-2. To visualize tumors, AR-2 was diluted in PBS, filtered through a 0.2 µm porous membrane, and intravenously injected via the lateral vein at 0.5 mg/kg *b.w.* bolus. The animals were then imaged 48 h after injection with a Pearl Impulse Imager (LI-COR Biosciences, NE) using the 800 nm channel. The fluorescence signals from acquired images were analyzed using Pearl Impulse Software v 2.0 (LI-COR Biosciences) according the manufacturer's protocols. For ATX inhibition studies, PF-8380 was suspended in a hydroxyethyl cellulose vehicle at 2 g/L. Mice were given PF-8380 (30 mg/kg, *b.w.*) or vehicle orally 20 min prior to AR-2 injection. ATX-Red dosing, delivery and imaging were unchanged.

### *Ex vivo* tissue analysis

Animals were sacrificed, organs were dissected and weighed, then *ex vivo* fluorescence was imaged and quantified as previously described. Tumors were immediately frozen until further use. Thawed tumors were homogenized in ice cold buffer containing 10 mM Tris pH 7.4, 200 mM NaCl, 10% glycerin, 5 mM EDTA and 1× Halt protease inhibitor cocktail (Thermo Scientific, Rockford, Il.) at a ratio of 40 mg tumor to 1 mL buffer. Samples were centrifuged at 14,000× *g* at 4°C. ATX levels were determined by diluting supernatants ten times in RD5-10 and assayed using a Human ENPP-2/Autotaxin ELISA (R&D Systems, Minneapolis, MN) and an Autotaxin Sandwich ELISA (Echelon Biosciences, Inc., Salt Lake City, UT) using the manufacturers' protocol. Total protein levels were determined by the method of Bradford [42]. Statistical significance was compared using a two-tailed t-test using Prism.

### Pharmacokinetics

To investigate preliminary PK of AR-2, female BALB/c mice aged 8–10 wks old (Charles River Laboratories, MA) were used. The animals were allowed to acclimatize to housing at least one week prior to use. At day 0, AR-2 was administered as a single bolus injection through lateral tail vein at a dosage of 1.0 mg/kg *b.w.* The animals were then lightly anesthetized with isoflurane for blood collection at 0, 0.08, 0.25, 0.5, 1, 2, 4, 6, 24, and 48 h after AR-2 administration. Blood samples were collected in EDTA coated tubes by submandibular bleeding or caudal vena cava (at time of sacrifice) with the maximum number of two blood collections from each animal. The collected blood samples were centrifuged, and the plasma was stored at −20°C until analysis.

To determine the concentration of AR-2 in plasma, samples were prepared according to the following procedures. Plasma samples were mixed with nine volumes of *tert*-butanol∶water (7∶1) then centrifuged at 14,000× *g* to pellet precipitated proteins. The resulting supernatant was frozen and lyophilized. Samples were then reconstituted in cold PBS and subjected to HPLC analysis using a C18 column (Waters, Milford, MA) with a gradient of 5–60% acetonitrile in 50 mM triethylammonium acetate (pH 6.0). Peaks with absorbance at 778 nm were integrated and compared to mouse plasma samples containing known concentrations of AR-2. The resulting AR-2 concentration versus time data were plotted and fitted with a sum of two exponentials to calculate plasma half-life (t_1/2_) and various pharmacokinetic parameters using the PK package in R Statistics (version 2.15.1).

## Supporting Information

Figure S1 **Pharmacokinetics of AR-2.** The time course of AR-2 in the plasma was fit to a bi-exponential equation yielding half-lives of 0.3 h and 8.5 h, respectively.
(TIF)

Figure S2 **AR-2 Synthesis. All reagents were purchased from Aldrich or Acros and used without further purification.** The NHS-esters of IRDye® QC-1 and IRDye® 800CW were purchased from Li-COR Biosciences. Chromatography was performed on an Isco Combiflash Companion using pre-packed C18 silica columns (Teledyne-Isco). ^1^H (400 MHz) and ^31^P (162 MHz) NMR spectra were recorded at 25°C on a Varian INOVA instrument. Chemical Shifts are given in ppm. Mass spectra were measured at the University of Utah Medicinal Chemistry Department using electrospray ionization (ESI). HPLC was performed on a Waters 2795 system with a 2990 diode array detector. The reverse phase column (NovaPak C18 4 µm, 3.9×150 mm) was eluted with a linear gradient of 0–40% acetonitrile in 50 mM triethylammonium bicarbonate (pH 6) over 15 min. *2*. Triethylamine (400 µL) was added to a solution of *1* ^24^ (39.7 mg, 45.2 µmol) and IRDye® QC-1 NHS ester (45 mg, 36.1 µmol) in dry DMF (12 mL) and the reaction was stirred for 2–3 hours protected from light. The solvents were evaporated under reduced pressure and the residue was co-evaporated twice with toluene. The product was purified by reverse phase chromatography using a linear gradient of, 5–40% acetonitrile in 50 mM triethylammonium acetate (pH 6.0). The desired fractions were pooled and dried under vacuum. Yield: 47.2 mg (58% as the triethylammonium salt). ^1^H NMR (CD_3_OD): 8.40 (d, *J* = 15.2 Hz, 1H), 8.25 (s, 1H), 8.13 (d, *J* = 8.8 Hz, 1H), 8.05 (d, *J* = 13.2 Hz, 1H), 7.89 (d, *J* = 9.2 Hz, 1H), 7.82 (d, *J* = 9.6 Hz, 1H), 7.37 (d, *J* = 8.4 Hz, 2H), 7.13 (s, 1H), 6.83 (d, *J* = 8.8 Hz, 1H), 6.67 (d, *J* = 14.8 Hz, 1H), 5.84 (d, *J* = 13.6 Hz, 1H), 4.45 (m, 2H), 4.14 (dd, *J* = 2.4, 10.4 Hz, 1H), 3.88–4.00 (m, 4H), 3.63–3.82 (m, 4H), 3.41–3.63 (m, 12H), 3.38 (t, *J* = 5.2 Hz, 2H), 3.30 (t, *J* = 5.2 Hz, 2H), 3.20 (s, 12H), 2.84–2.94 (m, 4H), 2.78 (t, *J* = 7.2 Hz, 4H), 2.68 (m, 2H), 2.61 (m, 2H), 2.35 (t, *J* = 6.0 Hz, 2H), 2.15–2.26 (m, 4H), 2.12 (t, *J* = 7.2 Hz, 2H), 1.92–2.04 (m, 4H), 1.84–1.90 (m, 5H), 1.82 (s, 9H), 1.723 (m, 2H), 1.69 (s, 3H), 1.44–1.61 (m, 4H), 1.27–1.43 (m, 10H), 0.78 (s, 9H), 0.01 (s, 3H), 0.00 (s, 3H). ^31^P NMR (CD_3_OD): 1.29. ESI-MS: 924.6 (M-2H)^2−^, 616.4 (M-3H)^3−^, 462.1 (M-4H)^4−^. *AR-2*. Trifluoroacetic acid (6 mL) was added to a solution of *2* (47.1 mg, 20.9 µmol) in CH_2_Cl_2_ (20 mL) and stirred for 1 hour in the dark. The solvents were evaporated under reduced pressure then co-evaporated with toluene (2×10 mL),1∶1 MeOH∶toluene (10 mL). The residue was dissolved in dry DMF (6 mL) then IRDye®800CW-NHS ester (31.4 mg, 26.9 µmol) was added followed by followed by triethylamine (250 µL). The reaction was stirred in the dark for 2–3 hours then the solvent was evaporated under reduced pressure. The product was purified by reverse phase chromatography using a linear gradient of, 5–40% acetonitrile in 50 mM triethylammonium acetate (pH 6.0). The desired fractions were pooled and dried under vacuum. Yield: 32 mg (50% as the triethylammonium salt). ^1^H NMR (CD_3_OD): 8.49 (d, *J* = 15.2 Hz, 1H), 8.35 (s, 1H), 8.23 (d, *J* = 8.8 Hz, 1H), 8.15 (d, *J* = 13.2 Hz, 1H), 7.76–8.07 (m, 10H), 7.48 (d, *J* = 8.4 Hz, 2H), 7.36 (d, J = 8.8 Hz, 1H), 7.30 (d, *J* = 8.4 Hz, 1H), 7.23 (s, 1H), 7.19 (d, *J* = 9.2 Hz, 2H), 6.93 (d, *J* = 8.8 Hz, 1H), 6.77 (d, *J* = 14.8 Hz, 1H), 6.08 (d, J = 14 Hz, 1H), 6.20 (d, J = 14.4 Hz, 1H), 5.94 (d, *J* = 13.6 Hz, 1H), 4.55 (m, 2H), 4.02–4.20 (m, 6H), 3.82–4.00 (m, 4H), 3.50–3.72 (m, 14H), 3.47 (t, *J* = 5.2 Hz, 2H), 3.40 (t, *J* = 5.2 Hz, 2H), 3.43 (s, 24H), 2.86–3.04 (m, 4H), 2.68–2.82 (m, 12H), 2.45 (t, *J* = 6.0 Hz, 2H), 2.25–2.36 (m, 4H), 1.10–2.26 (m, 45H). ^31^P NMR (CD_3_OD): 1.64. ESI-MS: 654.8 (M-4H)^4−^, 523.6 (M-5H)^5−^, 436.5 (M-6H)^6−^, 373.7 (M-7H)^7−^. HPLC 11.03 min (95% at 778 nm).
(TIF)
