# Supplementary figures and images for: Non-Invasive Imaging of Tumors by Monitoring Autotaxin Activity Using an Enzyme-Activated Near-Infrared Fluorogenic Substrate
Source: PLoS One. 2013 Nov 20;8(11):e79065. doi: 10.1371/journal.pone.0079065 (PMC3835791; doi:10.1371/journal.pone.0079065)

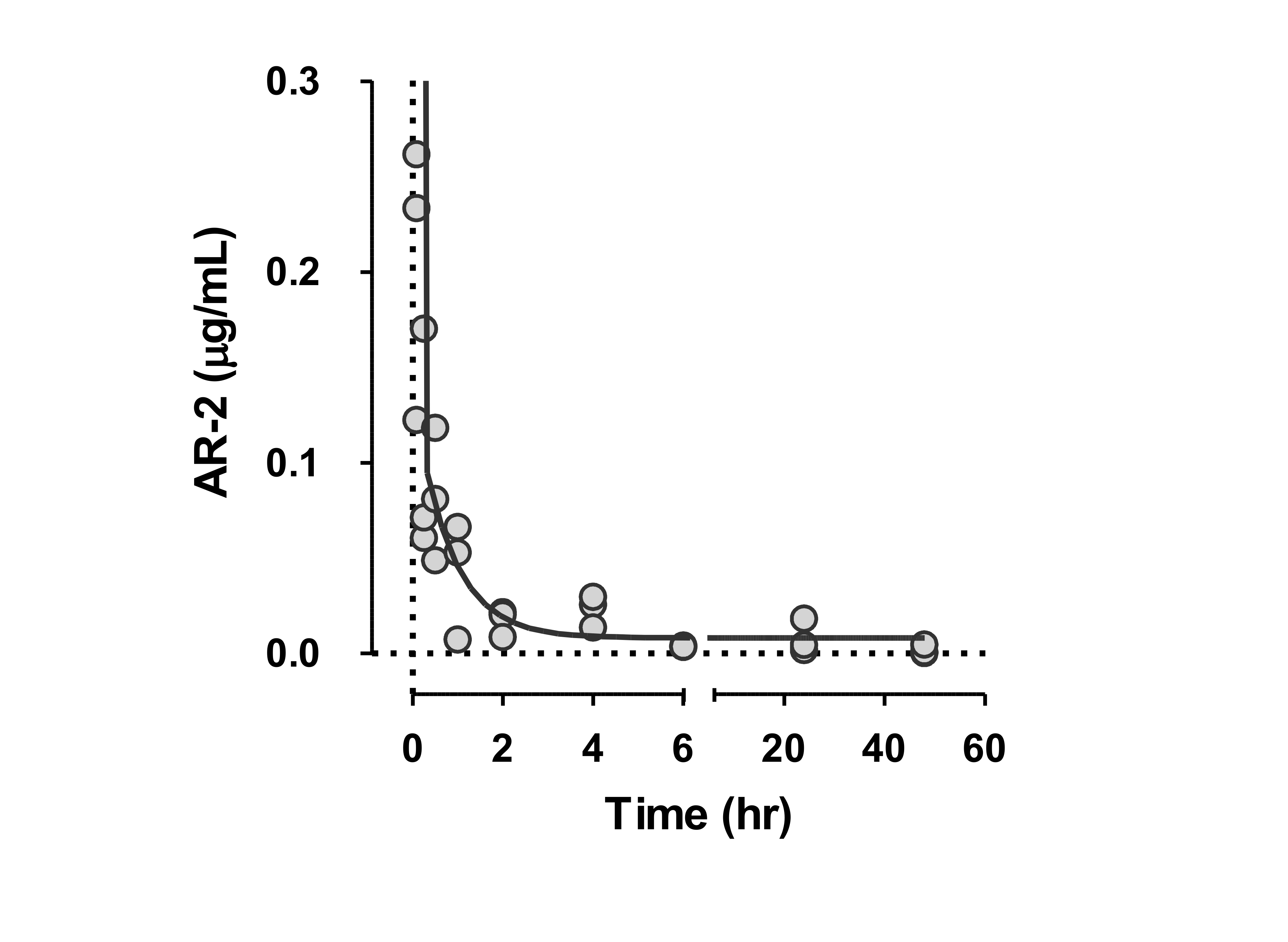

Supplement: Figure S1 — Pharmacokinetics of AR-2. The time course of AR-2 in the plasma was fit to a bi-exponential equation yielding half-lives of 0.3 h and 8.5 h, respectively. (TIF) [file pone.0079065.s001.tif]

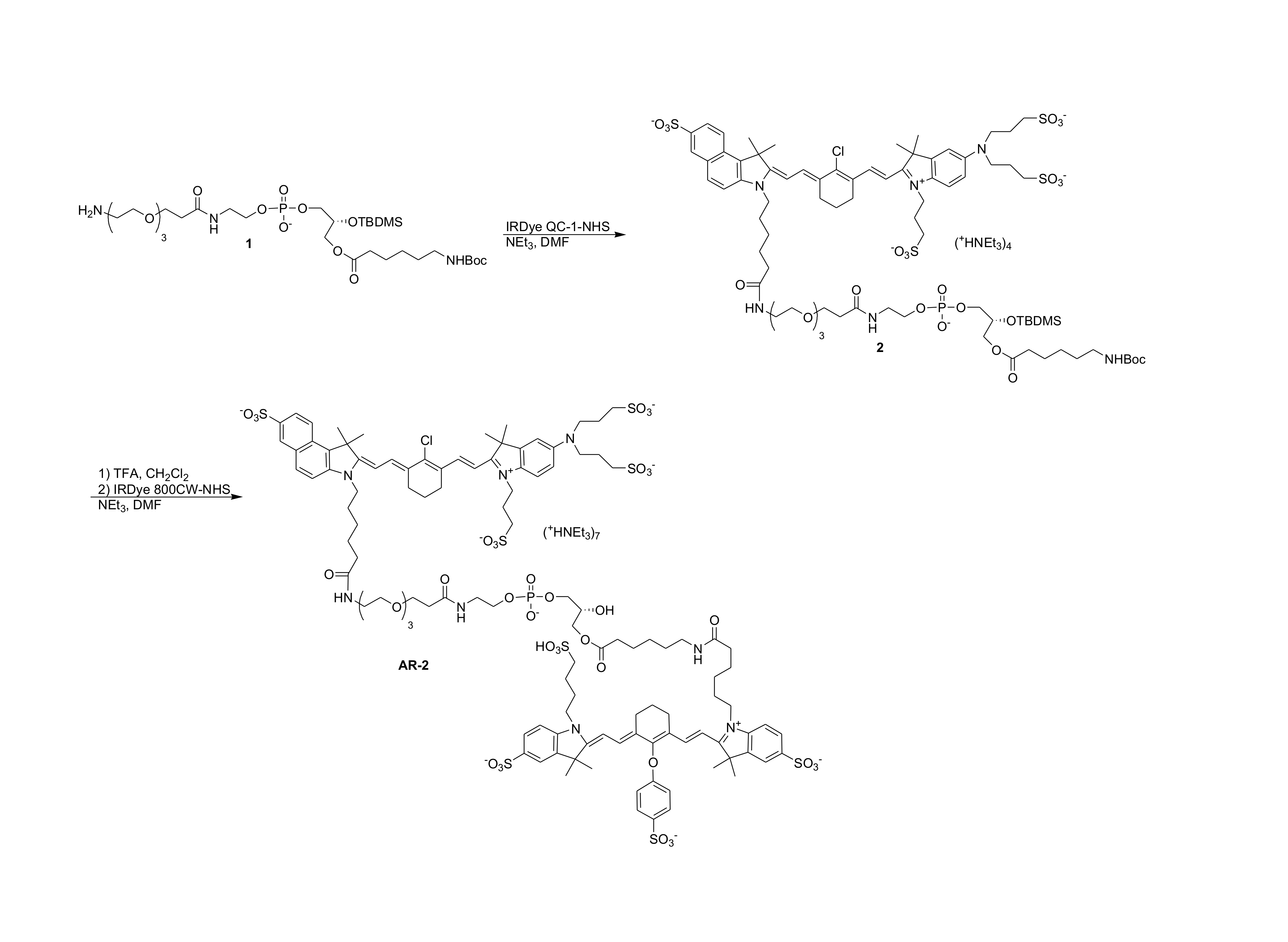

Supplement: Figure S2 — AR-2 Synthesis. All reagents were purchased from Aldrich or Acros and used without further purification. The NHS-esters of IRDye® QC-1 and IRDye® 800CW were purchased from Li-COR Biosciences. Chromatography was performed on an Isco Combiflash Companion using pre-packed C18 silica columns (Teledyne-Isco). 1H (400 MHz) and 31P (162 MHz) NMR spectra were recorded at 25°C on a Varian INOVA instrument. Chemical Shifts are given in ppm. Mass spectra were measured at the University of Utah Medicinal Chemistry Department using electrospray ionization (ESI). HPLC was performed on a Waters 2795 system with a 2990 diode array detector. The reverse phase column (NovaPak C18 4 µm, 3.9×150 mm) was eluted with a linear gradient of 0–40% acetonitrile in 50 mM triethylammonium bicarbonate (pH 6) over 15 min. 2. Triethylamine (400 µL) was added to a solution of 1 24 (39.7 mg, 45.2 µmol) and IRDye® QC-1 NHS ester (45 mg, 36.1 µmol) in dry DMF (12 mL) and the reaction was stirred for 2–3 hours protected from light. The solvents were evaporated under reduced pressure and the residue was co-evaporated twice with toluene. The product was purified by reverse phase chromatography using a linear gradient of, 5–40% acetonitrile in 50 mM triethylammonium acetate (pH 6.0). The desired fractions were pooled and dried under vacuum. Yield: 47.2 mg (58% as the triethylammonium salt). 1H NMR (CD3OD): 8.40 (d, J = 15.2 Hz, 1H), 8.25 (s, 1H), 8.13 (d, J = 8.8 Hz, 1H), 8.05 (d, J = 13.2 Hz, 1H), 7.89 (d, J = 9.2 Hz, 1H), 7.82 (d, J = 9.6 Hz, 1H), 7.37 (d, J = 8.4 Hz, 2H), 7.13 (s, 1H), 6.83 (d, J = 8.8 Hz, 1H), 6.67 (d, J = 14.8 Hz, 1H), 5.84 (d, J = 13.6 Hz, 1H), 4.45 (m, 2H), 4.14 (dd, J = 2.4, 10.4 Hz, 1H), 3.88–4.00 (m, 4H), 3.63–3.82 (m, 4H), 3.41–3.63 (m, 12H), 3.38 (t, J = 5.2 Hz, 2H), 3.30 (t, J = 5.2 Hz, 2H), 3.20 (s, 12H), 2.84–2.94 (m, 4H), 2.78 (t, J = 7.2 Hz, 4H), 2.68 (m, 2H), 2.61 (m, 2H), 2.35 (t, J = 6.0 Hz, 2H), 2.15–2.26 (m, 4H), 2.12 (t, J = 7.2 Hz, 2H), 1.92–2.04 (m, 4H), 1.84–1.90 [file pone.0079065.s002.tif]
